# Tractography Activation Patterns in Dorsolateral Prefrontal Cortex Suggest Better Clinical Responses in OCD DBS

**DOI:** 10.3389/fnins.2015.00519

**Published:** 2016-01-19

**Authors:** Christian J. Hartmann, J. Luis Lujan, Ashutosh Chaturvedi, Wayne K. Goodman, Michael S. Okun, Cameron C. McIntyre, Ihtsham U. Haq

**Affiliations:** ^1^Department of Biomedical Engineering, Cleveland Clinic FoundationCleveland, OH, USA; ^2^Department of Neurology, Medical Faculty, Institute of Clinical Neuroscience and Medical Psychology, Heinrich-Heine University DüsseldorfDüsseldorf, Germany; ^3^Department of Neurologic Surgery, Mayo ClinicRochester, MN, USA; ^4^Department of Physiology and Biomedical Engineering, Mayo ClinicRochester, MN, USA; ^5^Department of Biomedical Engineering, Case Western Reserve UniversityCleveland, OH, USA; ^6^Department of Psychiatry, Friedman Brain Institute and Mount Sinai School of MedicineNew York, NY, USA; ^7^Department of Neurology and Neurosurgery, Center for Movement Disorders and Neurorestoration, University of FloridaGainesville, FL, USA; ^8^Department of Neurology, Wake Forest University School of MedicineWinston-Salem, NC, USA

**Keywords:** obsessive-compulsive disorder, deep brain stimulation, tractography; simulation, clinical efficacy

## Abstract

**Background:** Medication resistant obsessive-compulsive disorder (OCD) patients can be successfully treated with Deep Brain Stimulation (DBS) which targets the anterior limb of the internal capsule (ALIC) and the nucleus accumbens (NA). Growing evidence suggests that in patients who respond to DBS, axonal fiber bundles surrounding the electrode are activated, but it is currently unknown which discrete pathways are critical for optimal benefit. Our aim was to identify axonal pathways mediating clinical effects of ALIC-NA DBS.

**Methods:** We created computational models of ALIC-NA DBS to simulate the activation of fiber tracts and to identify connected cerebral regions. The pattern of activated axons and their cortical targets was investigated in six OCD patients who underwent ALIC-NA DBS.

**Results:** Modulation of the right anterior middle frontal gyrus (dorsolateral prefrontal cortex) was associated with an excellent response. In contrast, non-responders showed high activation in the orbital part of the right inferior frontal gyrus (lateral orbitofrontal cortex/anterior ventrolateral prefrontal cortex). Factor analysis followed by step-wise linear regression indicated that YBOCS improvement was inversely associated with factors that were predominantly determined by gray matter activation results.

**Discussion:** Our findings support the hypothesis that optimal therapeutic results are associated with the activation of distinct fiber pathways. This suggests that in DBS for OCD, focused stimulation of specific fiber pathways, which would allow for stimulation with lower amplitudes, may be superior to activation of a wide array of pathways, typically associated with higher stimulation amplitudes.

## Introduction

Obsessive-compulsive disorder (OCD) is a common psychiatric disorder with a lifetime prevalence of 2.3% (Ruscio et al., 2010). It is defined by the presence of intrusive thoughts (obsessions) and the need to perform repetitive behaviors (compulsions) that severely impair quality of life (Leckman et al., 2010). While the pathogenesis of OCD has yet to be resolved, current hypotheses on the pathophysiology of OCD include abnormal activity in cortico-striatal-thalamo-cortical (CSTC) circuits (Aouizerate et al., 2004). Increased functional connectivity between cortical areas and the striatum has been observed in OCD patients (Sakai et al., 2011). Additionally, imaging studies in OCD patients have demonstrated baseline hyperactivity of orbitofrontal, prefrontal, and striatal areas, which increased further with symptom provocation (Bourne et al., 2012). While many OCD patients benefit from psychotherapy and drug treatment, 20–30% of patients do not respond to either form of therapy. Deep brain stimulation (DBS) of the anterior limb of the internal capsule (ALIC) and/or, nucleus accumbens (NA) presents a promising surgical alternative for these patients (Nuttin et al., 1999; Sturm et al., 2003; Okun et al., 2007; Goodman et al., 2010; Huff et al., 2010).

Little is known about the local and downstream effects of DBS despite its therapeutic efficacy. Recent studies in other disorders involving CSTC circuits (e.g., Parkinson's disease) have provided evidence that modulation of cortical activity is crucial for mediating the clinical effects of DBS (Walker et al., 2012a,b). This modulation may be driven by antidromic activation of corticofugal fibers projecting to (or passing by) the brain region where the DBS electrode is implanted (Li et al., 2007; Gradinaru et al., 2009). Furthermore, animal studies have demonstrated retrograde activation of inhibitory corticostriatal fibers in chronic high frequency (e.g., 130 Hz) DBS of the NA (McCracken and Grace, 2007). Nuclear imaging techniques have shown that prefrontal and orbitofrontal cortical metabolism decreases during therapeutic OCD DBS, similar to metabolic changes observed during pharmacotherapy or behavioral treatment (Swedo et al., 1992; Nuttin et al., 2003; Van Laere et al., 2006). Unfortunately, these imaging techniques are unable to distinguish between effects arising from direct modulation of connected white matter pathways (direct effects) and those resulting from compensatory network mechanisms (indirect effects). A detailed understanding of the interdependency of both phenomena will be a prerequisite for anatomically identifying preferential stimulation targets.

Recently our group developed the computational infrastructure for analyzing the network effects of DBS with tractography-activation models (TAMs; Lujan et al., 2012). TAMs combine tractography with neurostimulation modeling to simulate the brain pathways directly activated by patient specific DBS parameter settings. This tool can be used to specify the cerebral distribution of axonal activation and visualize the first line of neuronal response to DBS (Lujan et al., 2013). The results from this activation analysis can be translated into anatomical heat maps, which define the regional density of active fibers and offer an improved inter- and intra-individual comparability of activation results (Hartmann et al., 2015). The present study extends the use of TAMs to identify specific patterns of axonal activation in a case series of OCD patients treated with ALIC-NA DBS.

## Materials and methods

### Patient population

We used TAMs to analyze a cohort of six OCD patients who underwent bilateral DBS of the ALIC-NA region. All patients received pre- and post-operative psychiatric evaluations as part of a larger clinical trial using DBS for OCD (Goodman et al., 2010; Haq et al., 2011). We classified patients into three distinct groups according to their clinical response to DBS 24 month after surgery: best response, moderate response, and no-response. Best response was defined as having a minimum of 50% reduction in the Yale-Brown Obsessive Compulsive Scale (YBOCS, Goodman et al., 1989) scores compared to baseline. No-response was defined as a reduction of less than 10% in YBOCS scores. Patients who did not fulfill either criterion were regarded as moderate responders. Pertinent clinical data are summarized in Table 1. Ethical approval from the corresponding institutional review board (University of Florida) and informed written consent from the patients were obtained prior to their participation in this study.

**Table 1:** **Patient characteristics and DBS settings applied following 24 months of chronic ALIC-NA DBS**.

**#**	**Gender**	**Age at surgery**	**OCD symptoms**	**Response to DBS (YBOCS change)**	**DBS contacts and voltage**		**DBS frequency and pulse width**
					**Right side**	**Left side**	
1	F	33	Con Was Avo Anx	Best (68%)	1− C+, 5.0 V	0− C+, 5.0 V	135 Hz, 210 μs
2	M	52	Dou Avo Che	No (−3%)	1−2− C+, 4.0 V	1−2− C+, 1.5 V	135 Hz, 90 μs
3	M	39	Dou Che	Little (28%)	0−1− C+, 8.0 V	0−1− C+, 8.5 V	135 Hz, 150 μs
4	M	33	Con^*^	No (5%)	0−1−2− C+, 3.5 V	0−1−2− C+, 3.0 V	60 Hz, 180 μs
5	F	33	Con Avo Anx	Little (33%)	0−1− C+, 3.0 V	0−1− C+, 3.0 V	135 Hz, 210 μs
6	F	27	Con Avo Anx Was Cou	Best (86%)	1− 0+, 3.5 V	1− 0+, 3.3 V	135 Hz, 90 μs

Response to DBS is shown as YBOCS improvement compared to pre-operative assessments. The contacts of the DBS electrode are labeled from 0 to 3 for both sides, where contact 0 is the most ventral contact. A negative value for patient 2 indicates impairment. A negative value for patient 2 indicates impairment. Patient 4 (^*^) also suffered from Tourette syndrome as comorbidity. The stimulation frequency of 60 Hz was applied after usage of 135 Hz for 1 year turned out not to be beneficial. Abbreviations: Anx, Anxiety; Avo, Avoiding; C, stimulator case; Che, Checking; Con, contamination obsessions; Cou, Counting; DBS, Deep Brain Stimulation; Dou, Doubts; Was, Washing; YBOCS, Yale-Brown Obsessive Compulsive Scale.

### Morphological component of the DBS models

We developed six patient-specific models of DBS, where each brain hemisphere was analyzed separately. Each model comprised of a patient's pre- and post-operative anatomical imaging data and stimulation settings. These images were then co-registered to a single diffusion tensor image (DTI) brain atlas, which was also the foundation for a 3D finite element DBS electric field model (Chaturvedi et al., 2010). These models were developed using the following three-step process. First, we used FSL (Jenkinson et al., 2012) to co-register the patient's pre-operative T1-weighted 1.5 Tesla magnetic resonance images (MRI, 0.469 × 0.469 × 1.2 mm voxel size) and post-operative computed tomography (CT, 0.684 × 0.684 × 1.5 mm voxel size) data. For this purpose, rigid co-registration of the patient-specific MRI and CT scans was performed using FMRIB's Linear Image Registration Tool (FLIRT). This co-registration, which relied on six degrees of freedom to allow for translation and rotation along the x, y, and z-axis, respectively, used the individual MRI space as reference space. Similarly, the structural 1.5 Tesla MRI (1.0 × 1.0 × 1.0 mm voxel size) and DTI (2.0 × 2.0 × 2.0 mm voxel size, 60 gradient directions, *b* = 1000 s/mm^2^) of the reference brain atlas (Oxford Centre for Functional MRI of the Brain, Oxford, UK) were co-registered using an up-scaled DTI (1.0 × 1.0 × 1.0 mm voxel size) as reference space. Second, non-cerebral tissue was removed from each data set using FSL's Brain Extraction Tool. MRI scans were aligned with the atlas imaging data employing an affine registration (FNIRT) with 12 degrees of freedom (allowing for translation, rotation, scaling and skewing along the x, y, and z-axis, respectively). The resulting transformation matrix was applied to the corresponding patient specific CT scan. Third, we seeded a 3391 DBS electrode (Medtronic, Minneapolis, MN) within the DTI brain atlas employing the post-operative CT (Hemm et al., 2009). The DBS electrode (diameter of 1.27 mm) consisted of four vertically aligned contacts with a length of 3 mm and an inter-contact distance of 4 mm.

### Biophysical components of the DBS model

We created six quasi-static finite element electric field models (one for each patient) to characterize the DBS voltage distribution in brain tissue (Butson et al., 2007). For each model, a multi-resolution finite element method (FEM) model was constructed using COMSOL 3.1 (Comsol Inc., Burlington, MA) and SCIRun/Bio-PSE (Scientific Computing and Imaging Institute, University of Utah, Salt Lake City, UT). Active electrode contacts were defined as a voltage source for monopolar stimulation, while the outer surface of the model was defined as a boundary condition connected to ground. The model incorporated an encapsulation sheath surrounding the DBS electrode with a thickness of 0.5 mm to account for electrode impedance levels of 750–1250 Ω. The specific impedance of the encapsulation sheath was derived to match the overall impedance on the model to the clinically measured impedance in the patient (typical value ~0.1 S/m) (Butson et al., 2006; Chaturvedi et al., 2013). We also included a voltage drop at the electrode-tissue interface resulting from charge transfers from the electrode to the tissue (Gimsa et al., 2005; Miocinovic et al., 2009). This voltage drop was determined to be 42% in *in-vitro* studies using human DBS devices (Chaturvedi et al., 2010).

Conductivity tensors σ_T_ were calculated at each DTI voxel to incorporate the non-homogenous anisotropic conduction characteristics of the brain (Tuch et al., 2001; Haueisen et al., 2002). Each conductivity tensor was calculated using a linear transform of the local diffusion tensor D according to:

$$\begin{array}{l} \sigma_{\text{T}}=\left(\sigma_{\text{e}}∕{\text{d}}_{\text{e}}\right)\text{D}, \end{array}$$

where σ_e_ is the effective extracellular conductivity and d_e_ is the effective extracellular diffusivity. These conductivity tensors were interpolated onto a variable-resolution mesh (26 × 36 × 33 cm) to ensure both high FEM accuracy and computational efficiency (Chaturvedi et al., 2010). To account for the electrode capacitance at the electrode-tissue interface, a nominal value of 6.6 μF was derived from previous experiments (Holsheimer et al., 2000; Butson and McIntyre, 2005; Merrill et al., 2005). This electrode capacitance and model impedance was incorporated into a simple RC-filter, and an ideal square-wave stimulation waveform was fed through it. The output of this filter mimicked the actual waveform produced in the tissue medium during voltage-controlled stimulation (Miocinovic et al., 2009; Chaturvedi et al., 2010). This filtered waveform was subsequently used in the NEURON simulations to determine axonal activation (see below).

### Tractography-activation models (TAM)

We used TAMs to analyze axonal activation evoked by patient-specific DBS within a common DTI framework (voxel size 2 × 2 × 2 mm) (Lujan et al., 2012; Hartmann et al., 2015). TAMs were created for each brain hemisphere by the following six-step process. First, we defined two seed regions (one for each brain hemisphere) by combining voxels surrounding the geometric center of active contacts (radius of 4 mm) from each patient-specific computational model. These voxels were placed within the corresponding brain hemisphere of the reference DTI by applying the same transformation matrix that was previously used for the imaging data. The combination of these volumes resulted in a seeding volume of 454 voxels for the left hemisphere and 440 voxels for the right hemisphere. Second, we employed the FSL toolbox to perform probabilistic tractography from each voxel within both seed regions. A Bayesian algorithm (bedpostX) was used to calculate up to two fiber directions per voxel. This was followed by application of the program probtrackX, which iteratively created the 1000 most likely individual streamlines using Euler's method (step length 0.5 mm, curvature threshold ±80°). Pathway reconstruction was terminated when pathways looped onto themselves, cerebrospinal fluid space was reached, or when the algorithm took more than 2000 steps per pathway. Third, we used a custom built clustering algorithm to assess the distances between the streamlines at five critical points along each fiber (fiber origin, first quartile, midpoint, third quartile, and fiber termination; Lujan et al., 2012). For a given streamline, we computed the root mean squared (RMS) distance from all other streamlines at the five critical points described above. If the RMS distance was larger than 10 mm (5 mm for the midpoint) from the corresponding point along all pathways, the streamline geometry was considered unique and excluded from further analysis. These distance values were obtained by performing a sensitivity analysis to provide a balance between distinct pathways and the number of grouped pathways identified. Fourth, we created multi-compartment models of myelinated axons (5.7 μm diameter, 0.5 mm internodal distance) for each fiber trajectory within each pathway (McIntyre et al., 2002; Lujan et al., 2012). This fiber diameter, which is relatively large for human central nervous system white matter (Liewald et al., 2014), was chosen to avoid underestimation of the spread of axonal activation. Fifth, we determined the extracellular voltages along each axon model by interpolating each patient specific electric field model onto the axon trajectories. Finally, we simulated axonal response to extracellular stimulation and identified active fibers, defined as fibers generating a propagating action potential, using NEURON (Hines and Carnevale, 1997).

### Analysis of axonal activation

Cortical and subcortical gray matter structures were segmented from the reference brain structural MRI (Table 2) using Freesurfer (Athinoula A. Martinos Center for Biomedical Imaging, Charlestown, MA) (Fischl et al., 2002). We also identified the geometrical distribution of axonal activation (in the form of a heat map) by calculating the number of active axon fibers intersecting each voxel of a 1 × 1 × 1 mm grid co-registered with the reference DTI. Similarly, the intersection of active fibers with the segmented cortical and subcortical structures was calculated using MATLAB (The Mathworks, Natick, MA). Patient-specific activations from each hemisphere were combined to obtain a heat map of bilateral ALCI-NA DBS. These heat maps represented the local density of active fibers in each voxel by using color (the higher the density, the higher opacity, and brightness), thereby facilitating visual and quantitative analyses of activation results. Further details of this method are presented elsewhere (Hartmann et al., 2015). Following identification of crucial gray matter targeted by active fibers, we used the probtrackX*2* function within the FSL toolbox (applying the same specifications as used in probtrackX) to perform a connectivity-based probabilistic tractography analysis to classify the anatomical projections from each voxel within the seed region to these targets (Behrens et al., 2003). In addition to a descriptive analysis of gray matter targets of active fiber projections, factor (principal component) analysis was performed to determine if these activation results could be explained by unobserved factors. Subsequently, stepwise linear regression was used to identify predictors for the YBOCS change among these unobserved factors. The density of active fibers for each heat map voxel across best and moderate responders was compared to that of the same voxel in each non-responder's heat map. Based on preliminary analyses, differences in the density of active fibers greater or equal to 20 per voxel were empirically rated as relevant. The sum of voxels in all of the eight comparisons, which exhibited a relevantly higher density of active fibers in the responder, was correlated with the degree of clinical improvement (% of YBOCS) of this responder. Statistical analyses were performed with SPSS Statistics Version 20 (IBM Corp., Armonk, NY).

**Table 2:** **Overview of targeted cortical and subcortical regions and association with the degree of clinical response**.

	**Largest number of connecting active fibers**	**Smallest number of connecting active fibers**
Best response	Right middle frontal gyrus (anterior part)	Right accumbens area
		Right Amygdala
		Right superior frontal gyrus
		Right temporal lobe
Moderate response	Left superior frontal gyrus	Right Thalamus
		Right middle frontal gyrus (anterior part)
No response	Right Thalamus	–
	Right inferior frontal gyrus (orbital part)	

## Results

**Figure 1:**
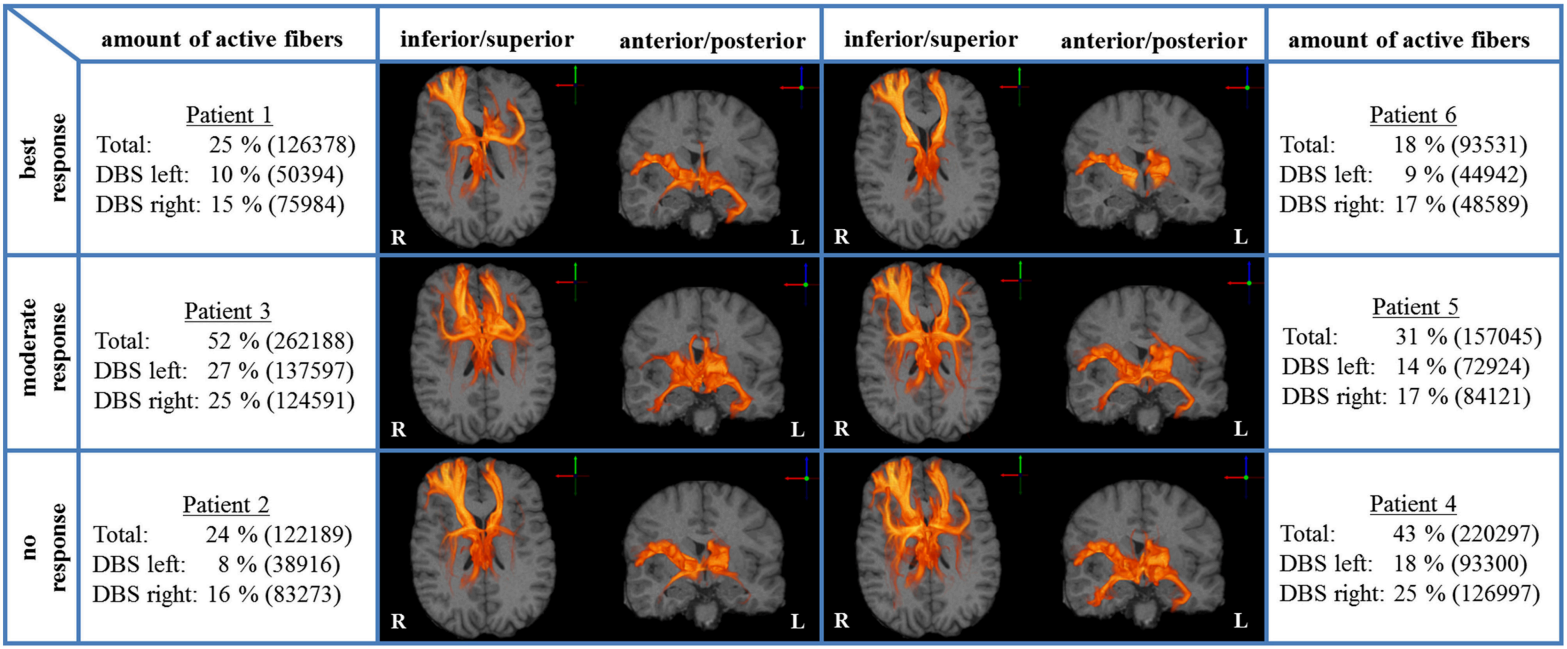
**Axonal activation heat maps (R, right; L, left)**. Increased brightness indicates a larger number of active fibers passing through a voxel. The amount of active fibers is defined as a percentage of all axon fibers investigated (absolute numbers of active fibers are provided in brackets). The patients were grouped based on the degree of clinical response (best, moderate, no response). All six patient-specific models show a similar anatomical distribution of active fibers.

We analyzed bilateral axonal activation in a group of six patients with OCD who underwent DBS of the ALIC-NA region. Patients 1 and 6 were classified as best responders with a reduction in YBOCS scores of 68 and 86% from baseline, respectively. Patients 2 and 4 were classified as non-responders with an increase of 3% and a reduction of 5% from baseline, respectively. The remaining two patients (3 and 5) were regarded as moderate responders with a reduction in YBOCS scores of 28 and 33% from baseline (Table 1). The individual activation results of the six patients including the heat maps indicating the distribution of active fibers are shown in Figure 1.

### Gray matter targets of active fiber projections

Among all axonal activation results (Table 2), non-responders (patients 2 and 4) showed the largest number of active fibers projecting to the right thalamus and orbital segment of the right inferior frontal gyrus. The group of patients with moderate clinical response (patients 3 and 5) presented with the least amount of active fibers targeting the right thalamus and the anterior portion of the right middle frontal gyrus. In contrast, this group showed the largest number of active fibers associated with the left superior frontal gyrus. Analysis of the two best responders (patients 1 and 6) revealed the least activation in the temporal lobe, superior frontal gyrus, amygdala, and the accumbens area of the right hemisphere. Furthermore, these patients possessed the most active fibers modeled to intersect with the right rostral middle frontal gyrus. Further details can be obtained in the supplementary material (Supplementary Table 1). Factor analysis identified four factors with an eigenvalue larger than 2, which were able to explain 96% of the observed variance among cortical activation results. Step-wise linear regression revealed that two of these factors were suitable to predict the observed YBOCS change in our patient cohort (adjusted *R*2 = 0.737). The first factor (Beta = −0.777) was highly correlated (correlation coefficient > 0.9) to the activation results of the right putamen. The second factor (Beta = −0.488) predominantly correlated (correlation coefficient > 0.9) with the orbitofrontal and cingulate cortices of both hemispheres as well as the right NA and the right caudate. Further details on factor and regression analysis are provided as supplementary material. Table 2 provides an overview of distinct cortical and subcortical regions targeted by active fibers, which are associated with the degree of clinical response. Association was assumed if both of the TAMs of a certain response group (best, moderate, no response) showed either highest or lowest number of active fibers in a distinct anatomical area.

### Parcellation of the seed region for tractography

**Figure 2:**
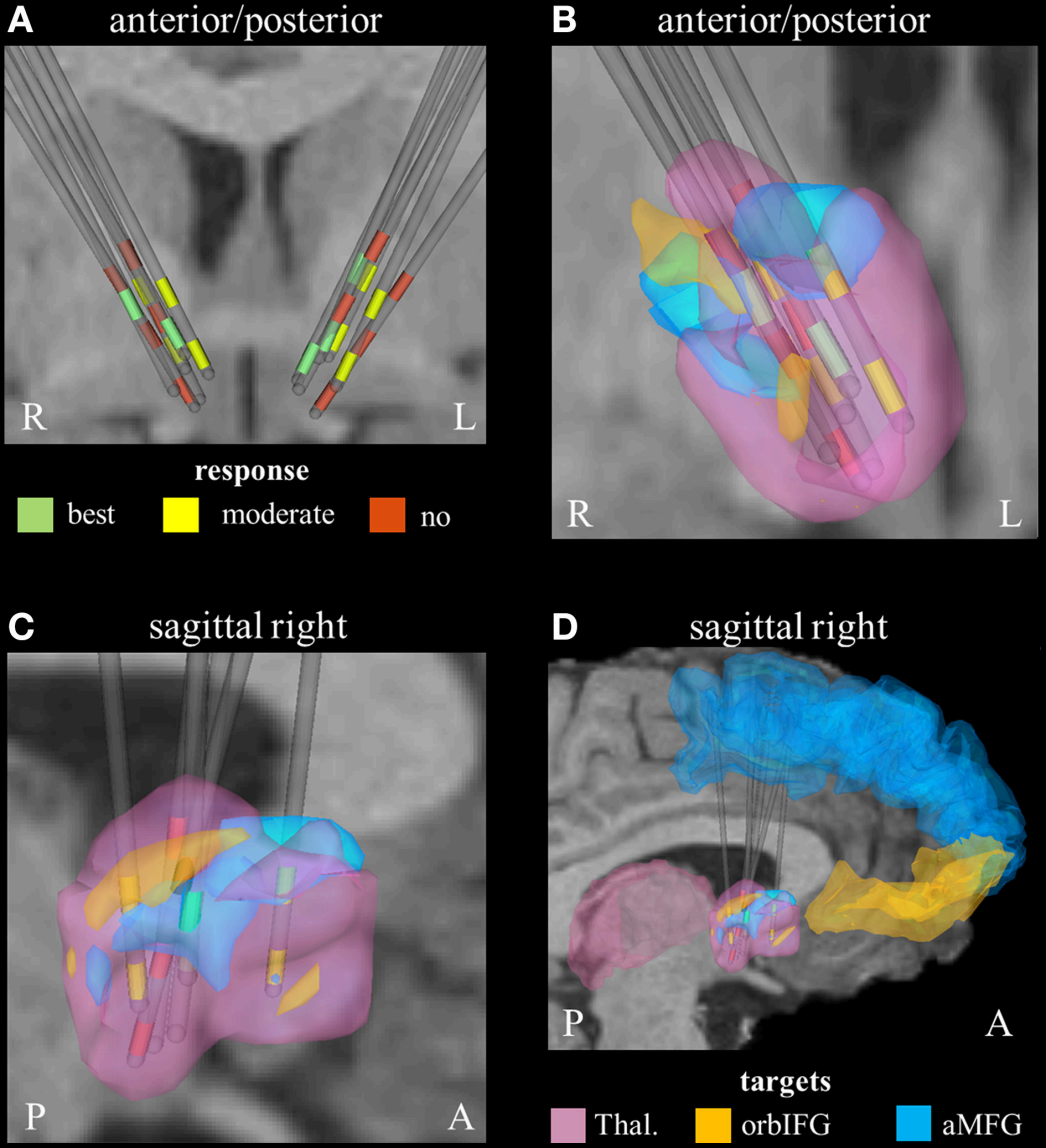
**Connectivity-based segmentation of the seed region for probabilistic tractography based on crucial gray matter targets of active fibers (R, right; L, left; P, posterior; A, anterior). (A)** Coronal view of the relative electrode position. The electrodes are labeled by the contact color (only active cathodes are shown). Green contacts represent electrodes of best responders, yellow contacts are assigned to moderate response, and red contacts correspond to non-responders. On the right hemisphere, the localization of dorsal active contacts in the non-responders is slightly superior compared to responders. Zoomed coronal **(B)** and sagittal **(C)** view of the right hemispheric seed region for tractography, which is classified based on predominant projection to three crucial areas: (1) The seed region with predominant projection to the right thalamus is shown in pink color. (2) The regions preferentially targeting the orbital part of the inferior frontal gyrus and the anterior part of the middle frontal gyrus on the right hemisphere are illustrated in orange and blue, respectively. (3) The uppermost contacts of the non-responders are closely related to parts of the seed region that preferentially projected to the orbital part of the inferior frontal gyrus. **(D)** Sagittal view of the seed region (from right) also showing the position of crucial target areas (Thal., thalamus; orbIFG, orbital part of the inferior frontal gyrus; aMFG, anterior part of the middle frontal gyrus).

Derived from the findings of the previous section, we identified three fundamental gray matter targets associated with either best or no response. The best response was found in connection with the right anterior part of the middle frontal gyrus. Non-responders were associated with projections to the thalamus and orbital part of the inferior frontal gyrus (right). Parcellation of the seed region for tractography revealed that most voxels were predominantly connected to the right thalamus. The anterior part of the right middle frontal cortex was predominantly represented in the center of the seed region. Voxels showing the highest connectivity to the orbital part of the right inferior frontal cortex were predominantly localized in the superior-lateral parts of the seed region (aside from a small portion in the anterior-inferior part, Figure 2).

### Subcortical targets of active fiber projections

**Figure 3:**
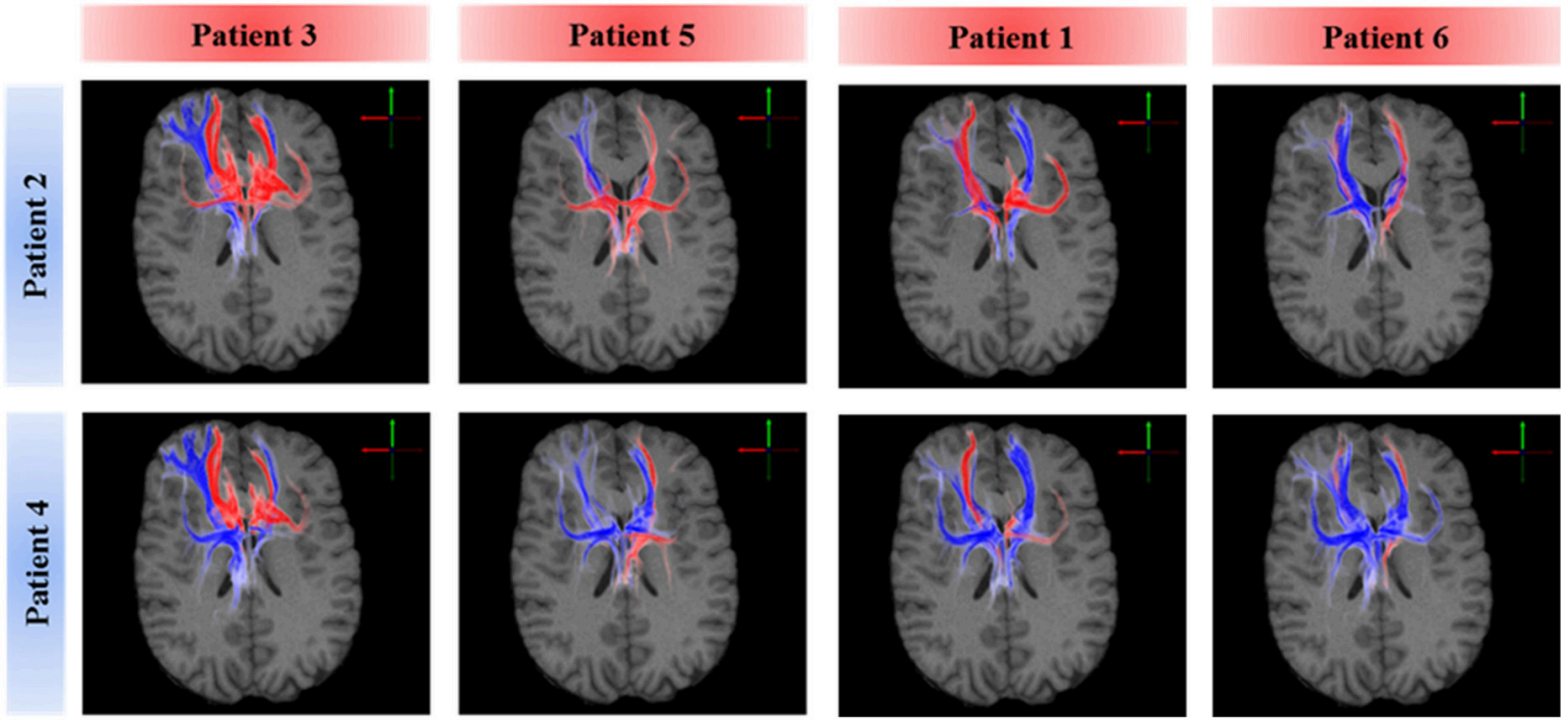
**Comparison of axonal activation between responders (columns) and non-responders (rows)**. Blue indicates predominant activation in a non-responder patient. Red indicates predominant activation in a responder patient. The spread of active fibers in responders is inversely correlated to DBS efficacy. Non-responders showed higher activation in the right orbital part of the inferior gyrus, which can be attributed to the lateral orbitofrontal cortex or the anterior ventrolateral prefrontal cortex.

A voxel-wise density analysis of the active fibers was performed for non-responders (patients 2 and 4) and responders (patients 1, 3, 5, and 6). Results of the comparison between responders and non-responders are shown as heat maps in Figure 1. This analysis provided information on which subcortical brain areas were preferentially targeted by active fibers and whether different anatomical representations were associated with different clinical outcomes. The distribution of these voxels was localized and well defined in the best clinical response group. In contrast, the distribution became broader for those patients with moderate response (Figure 3).

## Discussion

All six ALIC-NA DBS models showed active pathways associated with subcortical nuclei, prefrontal cortex, and the temporal lobe regardless of clinical response classification (Figure 1). These findings of distal gray matter regions directly modulated by ALIC-NA DBS are consistent with brain regions previously associated with the pathophysiology underlying OCD (Haber and Rauch, 2010). However, when considering our best responders to the DBS therapy, the TAMs provide support for a hypothesis that activation associated with the right middle frontal gyrus may have an especially important role in dictating clinical outcome. This area can be assigned to the dorsolateral prefrontal cortex (dlPFC, Brodmann areas 9 and 46), which is associated with executive functions, such as maintaining or shifting sets in response to changing task demands (Bonelli and Cummings, 2007). In contrast, activation of fibers projecting to the right thalamus or the orbital part of the inferior frontal gyrus (Brodmann are 47), which can be allocated to the anterior ventrolateral prefrontal cortex (avlPFC) and the lateral orbitofrontal cortex (lOFC), was prominent in non-responders. Furthermore, the overall amount of fiber activation, especially in the right hemisphere, may be inversely correlated with therapeutic effect.

### Asymmetric effects

Previous studies have shown functional and structural imbalances between the two hemispheres (Cannistraro et al., 2007). Additionally, these studies have shown that right-sided capsulotomy and TMS of the dlPFC alone can successfully improve symptoms of OCD (Greenberg et al., 1997; Lippitz et al., 1997, 1999). Furthermore, exclusive DBS of the right NA has shown therapeutic success (Sturm et al., 2003; Huff et al., 2010). In line with these findings, our data shows unilateral differences across the three patient classifications. These differences were predominantly found on the right hemisphere, suggesting that appropriate DBS of the right hemisphere might be critical for achieving therapeutic results.

### Activation of target and non-target neural elements

Activation of fibers projecting to the right middle frontal gyrus was most prominent in best responders. This suggests that modulation of the right dlPFC might be crucial for achieving optimal therapeutic outcomes (Table 2). However the clinical outcomes observed in these patients cannot be solely explained by activation of fibers targeting this region. Quantitative analysis of axonal activation revealed similar intensities of active pathways projecting to the dlPFC between best responders and non-responders (Supplementary Table 1). In contrast, activation of fiber pathways targeting the avlPFC/lOFC was predominantly found in non-responders. This area is associated with response inhibition, task-set switching, and maintenance of compulsive behavior (Aron et al., 2004; Elliott et al., 2010; Milad and Rauch, 2012). Saxena et al. showed that the metabolism of the ventrolateral prefrontal and the orbitofrontal cortex is reduced by effective drug treatment in OCD patients (Saxena et al., 2002). As such, it might have an important role in the development of compulsive behavior. Our data suggest that extensive activation of fibers targeting the avlPFC/lOFC by ALIC-NA DBS may prevent clinical improvement.

On the right hemisphere, the uppermost active contacts of non-responders (contacts 2) were placed superior, compared to the active contacts of responders (contacts 0 and/or 1, Table 1). These upper contacts were closely related to parts of the seed region that preferentially projected to the right avlPFC/lOFC, which in turn would explain the higher degree of activation of non-responders in that particular region (Figure 2). These findings are in line with a study by Lehman et al. who recently demonstrated that fiber tracts associated with avlPFC/lOFC are arranged more superiorly in the ALIC-NA region compared to fiber tracts associated with the medial orbitofrontal or ventromedial prefrontal cortices (Lehman et al., 2011). Furthermore, quantitative analysis of active fibers projecting to the right thalamic region showed the largest activation in non-responders. Conversely, the lowest activation was found in patients who showed moderate response. These data suggest that achieving optimal therapeutic benefits may not only require modulation of the correct anatomical targets, but also a delicate balance of stimulation within those targets.

### Extensive activation of non-therapeutically relevant fibers targets carries negative effects

Our results suggest that extensive activation of the right avlPFC/lOFC and right thalamus may counteract potential improvements gained by therapeutic modulation of the right dlPFC. Additionally, best responders showed the lowest overall amount of fiber activation for the right hemisphere, especially in the right NA region and the right temporal lobe. Moreover, factor analysis followed by step-wise linear regression indicates that YBOCS improvement is inversely associated with factors that are predominantly determined by gray matter activation results of the right hemisphere. Indeed, highest clinical improvement (Table 1, patient 6) was achieved with DBS leading to the lowest fiber activation of both hemispheres (Figure 1). All these observations are in agreement with the hypothesis that extensive activation of non-therapeutically relevant fibers reduces beneficial effects. Such reduction in therapeutic efficacy would be particularly important in diseases like OCD, depression, and dystonia, where clinical benefits gained with DBS may not be apparent for weeks or months. One might even speculate that this lack of immediate clinical results could, in turn, motivate DBS programmers to select high stimulation amplitudes, although they might not be necessary to achieve therapeutic effects (Anderson and Ahmed, 2003). Therefore, our computational model poses the hypothesis that a lower, more focused stimulation, explicitly targeting pathways associated with right dlPFC may be more beneficial for OCD patients treated with ALIC-NA DBS.

### Differences in clinical manifestation

Another potential explanation for the different clinical outcomes, despite similar patterns of fiber activation, is the variability in clinical manifestation. The clinical OCD spectrum is heterogeneous, and different network systems are predominantly involved in different clinical manifestations (Mataix-Cols et al., 2004; Saxena et al., 2004). Distinct pathologies may be more likely to respond to DBS of very specific networks. In this case, the clinical presentation of the disease could serve as a guide for electrode implantation and stimulator programming. For example, networks modulated by ALIC NA DBS might effectively impact washing compulsions, as these were a characteristic manifestation in the best responders. Unfortunately, not enough data are currently available for a comprehensive subgroup analysis. Future studies should compare DBS outcomes across different OCD subtypes.

### Limitations

This study presents a computational approach to analyzing patient-specific DBS axonal activation. The technique presented herein is based on a reference DTI brain that ensures an affordable use of imaging resources. Our investigation focused on large fiber pathways, which can be assumed to show a low inter-patient variability. Therefore, inter-individual differences of gross fiber pathways identified with patient-specific probabilistic tractography were not taken into account. Nevertheless, the use of patient-specific DTI scans could help further increase the precision of the analysis presented herein. Co-registration of patient-specific structural imaging data with a standardized DTI is likely to reduce inter-patient variability. However, we consider it a pragmatic approach to estimating fiber activation evoked by DBS while keeping the computational demand needed to develop the activation density heat maps at a tolerable level. The small number of patients presented prevents rigorous statistical analysis of pathways active across both responders and non-responders. However, empirical analysis represents the starting point toward the development of novel hypotheses. Additionally, activation differences between the three clinical groups are small, and the functional relevance of these differences has yet to be proven. Thus, a larger clinical trial comparing ALIC-NA DBS clinical outcomes to TAM cortical activation predictions is required to validate these results. Finally, the results are limited by the realization that the contacts used to model activated tracts in the non-responders may have been simply the last DBS lead contacts selected. The clinical benefits of DBS programming for OCD are not evident in the acute setting. As such, the contacts selected at the last follow-up, and thereby those used to model activated tracts in the non-responders, may have been clinically sub optimal in the chronic setting when clinical benefits were evaluated.

### Conclusion

This study presents a non-invasive method for elucidating axonal pathways activated by DBS and for improving understanding of the underlying mechanisms of DBS. The computational modeling approach presented herein will be critical for mapping cortical and subcortical targets associated with therapeutic benefits and/or adverse effects. Following validation on a larger patient population, this model could help to predict the optimal location for DBS electrode placement, as well as the clinical effects of various stimulation settings during post-operative patient programming.

## Author contributions

CH, JL, AC, WG, MO, CM, and IH contributed to the conception and the design of the study, acquired, analyzed, and interpreted the data. CH and JL drafted the manuscript, AC, WG, MO, CM, and IH have critically contributed to the manuscript and revised the draft. All authors have approved the final content of the manuscript.

## Funding

This work was supported by the National Institutes of Health (NIH R01 NS047388; NIH R01 NS059736; NIH R01 MH102238).

### Conflict of interest statement

The authors declare that the research was conducted in the absence of any commercial or financial relationships that could be construed as a potential conflict of interest. Intellectual Property: Boston Scientific Neuromodulation (J. Luis Lujan, Ashutosh Chaturvedi, Cameron C. McIntyre). Equity Interest: Surgical Information Sciences Inc., Autonomic Technologies Inc., Neuros Medical Inc. (Cameron C. McIntyre). Paid Consultants: Boston Scientific Neuromodulation (Cameron C. McIntyre), Lundbeck, Medtronics (Ihtsham U. Haq). National Parkinson Foundation (Michael S. Okun). Research grants: German Academic Exchange Service (Christian J. Hartmann). NIH, NPF, Michael J. Fox Foundation, Parkinson Alliance, Smallwood Foundation, Bachmann-Strauss Foundation, Tourette Syndrome Association, UF Foundation (Michael S. Okun). Sponsored CME and educational activities on movement disorders in the last 36 months: PeerView, Prime, Quantia, Henry Stewart, Vanderbilt University (Michael S. Okun). Royalties for publications: Demos, Manson, Amazon, Smashwords, Books4Patients, Cambridge (movement disorders books) (Michael S. Okun).
