# Supplementary material for: Tractography Activation Patterns in Dorsolateral Prefrontal Cortex Suggest Better Clinical Responses in OCD DBS
Source: Front Neurosci. 2016 Jan 19;9:519. doi: 10.3389/fnins.2015.00519 (PMC4717315; doi:10.3389/fnins.2015.00519)
Supplement: Supplementary file 1 [file DataSheet1.DOCX]

***Supplementary Material***

**Tractography activation patterns in dorsolateral prefrontal cortex suggest better clinical responses in OCD DBS**

**Christian J. Hartmann^1,2ǂ^, J. Luis Lujan^3,4ǂ^, Ashutosh Chaturvedi^5^, Wayne K. Goodman^6^, Michael S. Okun^7^, Cameron C. McIntyre^5*^, and Ihtsham U. Haq^8^**

^1^ Department of Biomedical Engineering, Cleveland Clinic Foundation, Cleveland, OH 44195, USA

^2^ Institute of Clinical Neuroscience and Medical Psychology, Department of Neurology, Heinrich-Heine University Düsseldorf, Medical Faculty, 40225 Düsseldorf, Germany

^3^ Department of Neurologic Surgery, Mayo Clinic, Rochester, MN 55905, USA

^4^ Department of Physiology and Biomedical Engineering, Mayo Clinic, Rochester, MN 55905, USA

^5^ Department of Biomedical Engineering, Case Western Reserve University, Cleveland, OH 44106, USA

^6^ Department of Psychiatry, Friedman Brain Institute & Mount Sinai School of Medicine, New York, NY 10029, USA

^7^ Department of Neurology and Neurosurgery, Center for Movement Disorders and Neurorestoration, University of Florida, Gainesville, FL 32611, USA

^8^ Department of Neurology, Wake Forest University School of Medicine, Winston-Salem, NC 27157, USA

**^*^ Correspondence:** Cameron C. McIntyre, Department of Biomedical Engineering, Case Western Reserve University, 10900 Euclid Avenue, Cleveland, OH 44106, USA.

ccm4@case.edu.

**^ǂ^** equally regarded as first authors

1. **Supplementary Data**

## Factor analysis

The activation results of all 30 gray matter regions presented in Suppl. Table 1 were included into a factor analysis to determine if these activation results could be explained by unobserved factors.

| **Quantitative analysis of active fibers targeting distinct grey matter regions.** | | | | | | | | |
| --- | --- | --- | --- | --- | --- | --- | --- | --- |
| **Region** | **Target** | | **Patient 1** | **Patient 2** | **Patient 3** | **Patient 4** | **Patient 5** | **Patient 6** |
| **Prefrontal cortex** | Superior frontal gyrus | L | 35 | 660 | 791 | 546 | 1861 | 647 |
|  |  | R | 166 | 207 | 305 | 240 | 218 | 152 |
|  | Middle frontal gyrus  anterior part | L | 50 | 7576 | 2526 | 6736 | 12950 | 10190 |
|  |  | R | 25848 | 24641 | 10082 | 25606 | 18519 | 25611 |
|  | Middle frontal gyrus  posterior part | L | 0 | 5 | 0 | 2 | 20 | 11 |
|  |  | R | 4 | 5 | 2 | 6 | 5 | 3 |
|  | Inferior frontal gyrus  pars orbitalis | L | 1 | 47 | 20 | 17 | 1155 | 21 |
|  |  | R | 12312 | 17931 | 1321 | 20604 | 16268 | 12886 |
|  | Inferior frontal gyrus  pars triangularis | L | 6 | 76 | 41 | 49 | 629 | 105 |
|  |  | R | 66 | 822 | 25 | 715 | 855 | 68 |
|  | Inferior frontal gyrus  pars opercularis | L | 0 | 1 | 2 | 4 | 5 | 5 |
|  |  | R | 31 | 53 | 13 | 50 | 52 | 26 |
|  | Orbitofrontal gyri  and frontal pole | L | 40736 | 13601 | 104575 | 51874 | 21781 | 15448 |
|  |  | R | 29969 | 22893 | 84365 | 33486 | 20580 | 13575 |
|  | Cingulate | L | 5986 | 173 | 14019 | 4240 | 790 | 18 |
|  |  | R | 551 | 315 | 39448 | 9677 | 2282 | 6 |
| **Temporal lobe** | | L | 8422 | 895 | 8476 | 5676 | 5992 | 330 |
|  |  | R | 1138 | 1849 | 4596 | 11011 | 4669 | 233 |
| **Subcortical structures** | Accumbens | L | 19529 | 18903 | 80642 | 46935 | 20028 | 11645 |
|  |  | R | 13501 | 13915 | 77302 | 28729 | 17746 | 277 |
|  | Amygdala | L | 7858 | 561 | 3199 | 4795 | 5525 | 326 |
|  |  | R | 1275 | 1984 | 55941 | 6515 | 20447 | 97 |
|  | Thalamus | L | 2178 | 20770 | 15434 | 20558 | 27593 | 25232 |
|  |  | R | 29243 | 40574 | 17403 | 34136 | 25718 | 29982 |
|  | Pallidum | L | 621 | 10846 | 14615 | 22436 | 47606 | 20680 |
|  |  | R | 59465 | 55153 | 17407 | 69832 | 57837 | 37224 |
|  | Putamen | L | 9854 | 24092 | 67169 | 62764 | 50230 | 28688 |
|  |  | R | 67453 | 61944 | 45703 | 84453 | 65134 | 37714 |
|  | Caudate | L | 1029 | 28011 | 37702 | 35459 | 31910 | 37370 |
|  |  | R | 14707 | 8630 | 33971 | 9674 | 6266 | 7285 |
| **Suppl. Table 1.** The values in the table reflect the sum of active fibers intersecting with the target brain area in the left (L) and right (R) hemisphere. | | | | | | | | |

Factors were determined based on principal components analysis, and all factors with an Eigenvalue greater than 2 were extracted. A total of four factors were extracted, that accounted for 9.4 - 45 % of total variance, respectively. Cumulatively, 96.5 % of the total variance could be explained by those four factors (Suppl. Table 2).

| **Total Variance Explained** | | | | | | | | | |
| --- | --- | --- | --- | --- | --- | --- | --- | --- | --- |
|  | Initial eigenvalues | | | Extraction sums of squared loadings | | | Rotation sums of squared loadings | | |
|  | Total | % of Variance | Cumulative % | Total | % of Variance | Cumulative % | Total | % of Variance | Cumulative % |
| 1 | 13.795 | 45.984 | 45.984 | 13.795 | 45.984 | 45.984 | 11.493 | 38.311 | 38.311 |
| 2 | 7.709 | 25.695 | 71.680 | 7.709 | 25.695 | 71.680 | 7.346 | 24.485 | 62.797 |
| 3 | 4.623 | 15.410 | 87.090 | 4.623 | 15.410 | 87.090 | 6.250 | 20.833 | 83.629 |
| 4 | 2.820 | 9.401 | 96.491 | 2.820 | 9.401 | 96.491 | 3.859 | 12.862 | 96.491 |
| 5 | 1.053 | 3.509 | 100.000 |  |  |  |  |  |  |

**Supp. Table 2.** Details on the first five factors which were extracted from gray matter activation results. The first four factors have an Eigenvalue greater than 2 and were therefore kept for further analysis

The component matrix was investigated for gray matter activation results that were highly correlated with the associated factor. Values of +/- .9 were regarded as highly associated with the factor. Supp. Table 2 provides the raw data of the component matrix for the four different factors.

|  | Component | | | |
| --- | --- | --- | --- | --- |
|  | 1 | 2 | 3 | 4 |
| Superior frontal gyrus left | -.196 | .925 | -.064 | -.260 |
| Superior frontal gyrus right | .722 | .490 | .369 | .234 |
| Middle frontal gyrus anterior part left | -.641 | .713 | -.266 | .094 |
| Middlefrontal gyrus anterior part right | -.783 | -.545 | .075 | .164 |
| Middle frontal gyrus posterior part left | -.576 | .674 | -.311 | -.341 |
| Middle frontal gyrus posterior part right | -.711 | .081 | .681 | .147 |
| Inferior frontal gyrus pars orbitalis left | -.344 | .728 | .084 | -.572 |
| Inferiorfrontal gyrus pars orbitalis right | -.890 | -.003 | .396 | .220 |
| Inferior frontal gyrus pars triangularis left | -.390 | .760 | -.002 | -.509 |
| Inferiorf rontal gyrus pars triangularis right | -.615 | .455 | .481 | .183 |
| Inferior frontal gyrus pars opercularis left | -.327 | .744 | -.213 | .119 |
| Inferior frontal gyrus pars opercularis right | -.792 | .209 | .490 | .109 |
| Orbitofrontal left | .957 | .094 | .248 | .061 |
| Orbitofrontal right | .972 | .122 | .148 | .080 |
| Cingulate left | .977 | -.050 | .196 | -.065 |
| Cingulate right | .944 | .277 | .069 | .163 |
| Temporal lobe left | .625 | -.004 | .554 | -.536 |
| Temporal lobe right | .102 | .414 | .777 | .350 |
| Accumbens left | .887 | .254 | .290 | .253 |
| Accumbens right | .918 | .283 | .220 | .112 |
| Amygdala left | .105 | -.151 | .651 | -.698 |
| Amygdala right | .853 | .490 | .005 | -.089 |
| Thalamus left | -.415 | .796 | -.276 | .342 |
| Thalamus right | -.757 | -.369 | .166 | .454 |
| Pallidum left | -.330 | .915 | .025 | -.191 |
| Pallidum right | -.740 | -.162 | .646 | -.058 |
| Putamen left | .467 | .743 | .317 | .301 |
| Putamen right | -.326 | -.060 | .943 | .015 |
| Caudate left | .108 | .749 | -.245 | .594 |
| Caudate right | .994 | -.026 | -.001 | -.009 |

**Supp. Table 3.** Component matrix for the four extracted factors. All gray matter activation results that are highly associated with a given factor are highlighted.

## Regression analysis

Stepwise linear regression was used to identify predictors for the YBOCS change among the four extracted factors. Factors number 3 and 1 were kept, while factors 2 and 4 were removed from the model. The combination of factors 3 and 1 provided a better fit than a model solely employing factor 3 (Adjusted R Square = .737 vs .506). The coefficients of the models are provided in Suppl. Table 3.

| Model | | Unstandardized Coefficients | | Standardized Coefficients | t | Sig. | Correlations | | | Collinearity statistics | |
| --- | --- | --- | --- | --- | --- | --- | --- | --- | --- | --- | --- |
|  |  | B | Std. Error | Beta |  |  | Zero-order | Partial | Part | Tolerance | VIF |
| 1 | (Constant) | 36.167 | 10.009 |  | 3.613 | .022 |  |  |  |  |  |
|  | Factor 3 | -27.109 | 10.965 | -.777 | -2.472 | .069 | -.777 | -.777 | -.777 | 1.000 | 1.000 |
| 2 | (Constant) | 36.167 | 7.295 |  | 4.958 | .016 |  |  |  |  |  |
|  | Factor 3 | -27.109 | 7.991 | -.777 | -3.393 | .043 | -.777 | -.891 | -.777 | 1.000 | 1.000 |
|  | Factor 1 | -17.010 | 7.991 | -.488 | -2.129 | .123 | -.488 | -.776 | -.488 | 1.000 | 1.000 |

**Suppl. Table 4.** Coefficients of the regression models. B refers to the unstandardized regression coefficient, whereas bets refers to the standardized regression coefficient
